# Personal Strategies for DIEP Flap Breast Reconstruction in Patients with Prior Abdominal Surgery and Hernia Repairs

**DOI:** 10.1055/a-2710-4367

**Published:** 2026-01-30

**Authors:** Samarth Gupta, Rajan Arora, Kripa Mishra, Anchit Kumar, Nikhil Prasad

**Affiliations:** 1 Department of Reconstructive and Microvascular Surgery, Rajiv Gandhi Cancer Institute and Research Centre, New Delhi, India

**Keywords:** DIEP flaps in hernia, abdominal scars and DIEP, breast reconstruction

## Abstract

Delayed breast reconstruction using deep inferior epigastric perforator (DIEP) flaps in patients with a history of abdominal wall hernias and/or cesarean sections presents unique challenges. This study examines 10 such cases, emphasizing key technical considerations. Our findings highlight the importance of lateral row perforators, as medial paraumbilical perforators are often compromised in patients with prior umbilical hernia repairs. Additionally, deep inferior epigastric arteries (DIEAs) may be damaged in previous lower abdominal surgeries, necessitating intraoperative confirmation of vessel patency. While preoperative CT angiography aids in planning, it may misrepresent perforator size or location due to adherence to fascia. In our approach, a gastrointestinal surgeon performed concurrent hernia repair while the plastic surgery team secured the DIEP flap perforators and pedicle. Preservation of umbilical vascularity was ensured by avoiding complete skeletonization. In the case shown, only a single lateral row perforator was usable, despite preoperative imaging suggesting additional perforators. All patients had successful flap integration, with no cases of flap failure, necrosis, postoperative hernias, wound dehiscence, seroma, hematoma, or infection. A delayed flap inset was performed using the Rosebud technique, ensuring optimal aesthetic outcomes and high patient satisfaction. This study highlights the critical role of a multidisciplinary approach, precise perforator identification, and careful interpretation of preoperative imaging in achieving optimal outcomes in complex DIEP flap breast reconstruction.

Deep inferior epigastric perforator (DIEP) flap breast reconstruction in patients with a history of abdominal hernias and/or cesarean sections presents unique challenges. 1 Over the course of 10 DIEP cases in such patients, we have identified key considerations that influence surgical planning and outcomes. This paper highlights critical aspects of preoperative imaging, intraoperative strategies, and the importance of a multidisciplinary approach.

One of the most significant observations from our series is the importance of lateral row perforators. Medial paraumbilical perforators are often compromised in patients with prior umbilical hernia repairs, necessitating a shift in focus toward lateral row perforators for successful flap harvest. 2 Another critical factor is the evaluation of deep inferior epigastric arteries (DIEAs), as previous caesarean sections and lower abdominal wall hernias may damage or occlude these vessels.

Preoperative CT angiography is a valuable tool for mapping perforators and planning DIEP flap reconstruction, with studies such as Lam et al (2012) and Fitzgerald O'Connor et al (2016) demonstrating its role in reducing operative time and complications. 3 4 However, CT imaging has limitations, particularly in patients with prior abdominal surgeries, where it may not accurately predict perforator viability. Some vessels may be too small, adherent to the fascia, or otherwise unsuitable for dissection. Intraoperative confirmation using direct visualization, Doppler ultrasound, or indocyanine green fluorescence angiography is essential for real-time assessment of vascular flow and DIEA patency. If intraoperative challenges arise, alternatives such as contralateral perforators or other flaps—like the transverse upper gracilis or lumbar artery perforator flap—should be considered based on patient anatomy and preferences.

Preserving the vascularity of the umbilicus remains a challenge in these cases. To prevent ischemic complications, we recommend avoiding complete skeletonization and instead retaining adequate surrounding fat, even in non-complicated patients. This approach ensures a robust blood supply, reduces the risk of ischemia, and enhances aesthetic outcomes. Figure 1 showcases a patient with a history of paraumbilical hernia repair and lower abdominal wall hernia following two cesarean sections. In this case, demonstrated in Video 1 , only a single sizable lateral row perforator was viable on the left side, despite preoperative CT imaging indicating a usable perforator on the right. The 3D volume rendering technique (VRT) for Video 1 was performed using syngo.via software integrated with the SOMATOM Definition AS+ CT scanner (Siemens Healthineers). This case highlights the critical role of intraoperative decision-making and emphasizes the potential discrepancies between preoperative imaging and real-time surgical findings.

**Fig. 1:**
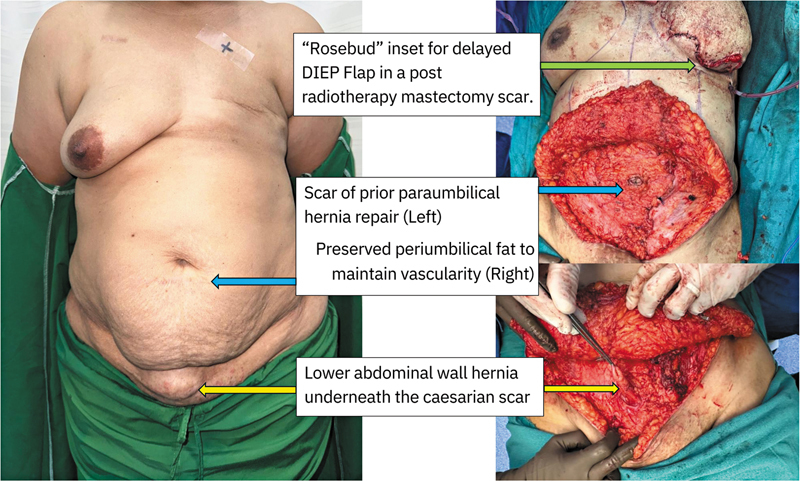
Intraoperative and preoperative images of a patient with prior paraumbilical hernia repair and cesarean sections.

**Video 1** Video demonstrating abdominal wall defects and lateral row perforator in a patient with previous abdominal surgeries, highlighting the importance of preoperative decision-making.


The outcomes of our series were highly favorable. None of the patients developed flap failure, flap necrosis, or postoperative abdominal wall hernias. We frequently use prophylactic venous supercharging to maintain venous outflow in more than half of our flaps. Additionally, patient satisfaction scores were excellent, reflecting both functional and aesthetic success. A delayed flap inset was performed using our previously published Rosebud technique, which has been instrumental in achieving optimal breast contouring. 5

Each case presented unique challenges that emphasized the importance of individualized surgical planning. Patients with multiple prior abdominal surgeries often have extensive scar tissue, making perforator dissection more complex. In three cases, the DIEA was partially compromised, necessitating opposite side harvest or adjustments in flap design to optimize perfusion.

An important consideration in our approach was donor site closure. 6 Given the history of prior surgeries, ensuring tension-free closure while maintaining abdominal wall integrity was paramount. The use of progressive tension sutures and careful layering of closure techniques helped prevent complications such as seromas or wound dehiscence. Despite the complexity of these cases, no donor site complications were observed in our series.

In cases with abdominal wall hernias, involving a gastrointestinal (GI) surgeon improves outcomes. In the case shown, the plastic surgery team secured the perforator before the GI team repaired the hernia with a 30 × 30 cm onlay Prolene mesh ( Fig. 2 ), providing durable support and reducing recurrence risk. This highlights the importance of a dual-team approach in complex DIEP flap reconstructions.

**Fig. 2:**
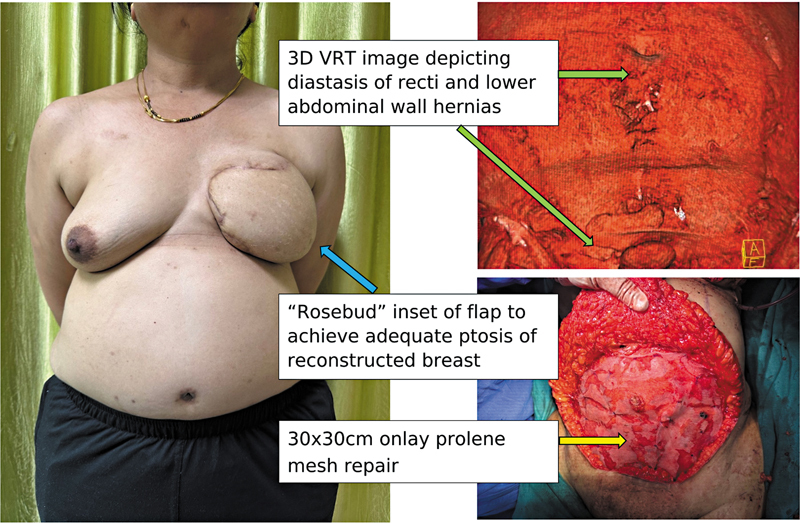
Onlay mesh reinforcement (30 × 30 cm) of the abdominal wall following hernia repair in a DIEP flap patient, providing durable support and minimizing recurrence risk. DIEP, deep inferior epigastric perforator; VRT, volume rendering technique.

A key finding in our series was the reliability of lateral row perforators in cases where medial perforators were compromised. In patients with extensive abdominal scarring, the shift in focus toward lateral row perforators ensured consistent flap viability. While CT angiography remains a useful preoperative tool, real-time intraoperative assessment remains the most reliable determinant of perforator suitability. Table 1 shows DIEP flap reconstruction in our series of patients with prior abdominal surgeries.

**Table 1:** Deep inferior epigastric perforator flap reconstruction in patients with prior abdominal surgeries

Patient	Previous surgery	Injured structures	Used perforators	Number of perforators	Flap weight (g)	Location and size of hernia	Follow-up period (months)	Complications	DIEA patency	Supercharging	Flap outcome
1	Umbilical hernia repair	Medial perforator compromised	Lateral	1	350 g	N/A	12	None	Patent	No	Healthy
2	Appendectomy	None	Medial	2	375 g	N/A	14	None	Patent	No	Healthy
3	Abdominal hysterectomy	Minor scarring near DIEA	Medial	1	400 g	None	10	None	Patent	No	Healthy
4	Cesarean section ×2	Medial perforators damaged, lateral damaged on one side	Lateral	1	400 g	Two Caesarian scar hernias; 4cm, 3 cm	10	None	Patent	Yes	Healthy
5	Umbilical hernia repair	Medial perforator adherent	Lateral	1	325 g	None	11	None	Patent	No	Healthy
6	Laparoscopic cholecystectomy	None identified	Medial	1	460 g	None	9	None	Patent	Yes	Healthy
7	Liposuction	Scarring around medial perforator	Lateral	2	390 g	None	13	None	Patent	Yes	Healthy
8	Ventral hernia repair	DIEA partial occlusion	Lateral	2	415 g	None	16	None	Patent	Yes	Healthy
9	Cesarean section, open appendectomy	DIEA minor scarring	Lateral	1	455 g	None	12	None	Patent	No	Healthy
10	Paraumbilical hernia repair	Medial perforator damaged	Lateral	1	380 g	None	10	None	Patent	No	Healthy

Abbreviations: DIEA, deep inferior epigastric artery.

Patient series of deep inferior epigastric perforator flap reconstruction in prior abdominal surgery and hernia repairs.

## Conclusion

DIEP flap reconstruction in patients with prior abdominal surgeries requires meticulous planning and a tailored approach. Emphasizing lateral row perforators, verifying vascular anatomy intraoperatively, and involving GI surgeons for concurrent hernia repair significantly improve outcomes. Understanding the limitations of CT imaging and preserving umbilical vascularity are also crucial elements of success. This multidisciplinary approach maximizes both reconstructive success and patient satisfaction, ultimately reducing the need for future corrective procedures.
